# Multifunctional Activity of *Lippia gracilis* Schauer Essential Oil Against Skin Infections

**DOI:** 10.3390/plants15111681

**Published:** 2026-05-29

**Authors:** Igor Lima Soares, Kellen Sá, Kirley Marques Canuto, Mary Anne Bandeira, Lígia Salgueiro, Mónica Zuzarte

**Affiliations:** 1 Postgraduate Program in Development and Technological Innovation in Medicines (PPgDITM), Federal University of Ceará (UFC), Fortaleza 60430-160, CE, Brazil; igorlima.ti@gmail.com (I.L.S.); mambandeira@yahoo.com.br (M.A.B.); 2 Medicinal Plants Garden Professor Francisco José de Abreu Matos, Federal University of Ceará (UFC), Fortaleza 60455-970, CE, Brazil; kellenmiranda@ufc.br; 3 Embrapa Tropical Agroindustry, Fortaleza 60511-110, CE, Brazil; kirley.canuto@embrapa.br; 4 Department of Pharmacy, Federal University of Ceará, Fortaleza 60430-370, CE, Brazil; 5 Faculty of Pharmacy, University of Coimbra, 3000-548 Coimbra, Portugal; ligia@ff.uc.pt; 6 Department of Chemical Engineering, Chemical Engineering and Renewable Resources for Sustainability (CERES), University of Coimbra, 3030-790 Coimbra, Portugal; 7 Coimbra Institute for Clinical and Biomedical Research, Centre for Innovative Biomedicine and Biotechnology (iCBR-CIBB), University of Coimbra, 3000-548 Coimbra, Portugal; 8 Faculty of Medicine, University of Coimbra, 3000-548 Coimbra, Portugal

**Keywords:** biofilm, cell migration, dermatophytes, *Epidermophyton floccosum*, *Trichophyton rubrum*, onicomycosis, Verbenaceae, thymol, wound healing

## Abstract

This study evaluates the antifungal activity of *Lippia gracilis* Schauer essential oil traditionally used in northeastern Brazil for the treatment of skin disorders. The essential oil isolated from fresh leaves collected in Ceará, Brazil, was chemically characterized by GC–MS, showing a thymol-dominant profile (37.52%), accompanied by *p*-cymene (12.13%), γ-terpinene (9.29%), and gurjunene (10.95%). Antifungal assays revealed significant activity against several dermatophyte species with MIC and MFC values ranging from 50 to 100 μg/mL. Biofilm assays against *Epidermophyton floccosum* demonstrated strong inhibitory effects by disrupting biofilm formation, altering fungal morphology, and promoting in vitro skin cells migration, while also showing effective preventive effects in an ex vitro nail infection model. These findings support the potential of *L. gracilis* essential oil as a multifunctional natural antifungal agent for the management of refractory dermatophytosis and onychomycosis, and provide scientific validation for its traditional use in skin infection treatment.

## 1. Introduction

Climate change has contributed to the rising incidence of cutaneous fungal infections and the emergence of strains resistant to conventional treatments, posing an increasing challenge to clinical practice and drug development [1,2,3]. Furthermore, zoonotic transmission of dermatophytes from animals such as cats, cattle, and small mammals to humans have been increasingly reported, highlighting the complex interplay between environmental changes and animal reservoirs [4,5].

The ability of dermatophytes to develop structured biofilms further intensifies this scenario, as these communities enhance fungal persistence under environmental stress and markedly reduce antifungal susceptibility [6]. Such biofilm-associated traits likely contribute to the maintenance and dissemination of resistant isolates in various ecological and clinical settings, thereby complicating therapeutic management and infection control strategies [7,8,9,10].

Dermatophyte biofilms not only confer multidrug resistance but also impair the penetration of antifungal agents, as the extracellular matrix acts as a physical barrier [11]. This phenomenon is particularly problematic for onychomycosis and tinea infections, where therapeutic failure rates remain high despite prolonged treatment regimens [12,13,14]. Consequently, dermatophytic infections of the skin and nails can compromise the integrity of the tissue by degrading keratin [15], sometimes leading to lesions or wounds that are difficult to heal and may serve as entry points for secondary infections [16].

This rising antifungal resistance, partly driven by the inappropriate and widespread use of antifungals and corticosteroids, constitutes an escalating global public health threat [17] and highlights the urgent need for the development of novel therapeutic alternatives, particularly from natural products [18,19,20,21]. For instance, essential oils exhibit antifungal activity due to synergistic interactions among multiple compounds acting on different cellular targets, thereby enhancing efficacy and reducing the likelihood of resistance [22,23].

Within this context, species from the genus *Lippia* emerge as particularly promising sources of bioactive compounds [24,25,26]. *Lippia* (Verbenaceae) comprises more than 200 species widely distributed in tropical and subtropical regions, known for their essential oils rich in mono- and sesquiterpenes [27]. Several species have been traditionally used for their antimicrobial and anti-inflammatory properties, which are often linked to chemical diversity and the presence of distinct chemotypes within this genus [28].

Among these, *Lippia gracilis* Schauer (syn. *Lippia grata* Schauer) is a neotropical species distributed mainly in South America, occurring from northern Argentina to northeastern Brazil, with records also in Bolivia and Venezuela [29]. In Brazil, it is found in the Northeast Region, particularly in the states of Bahia, Sergipe, Maranhão, Pernambuco, Paraíba, Piauí, Rio Grande do Norte and Ceará, and may also occur in parts of Minas Gerais within the Caatinga and Cerrado transition zones [30]. *L. gracilis* is commonly known as “alecrim-de-tabuleiro” (rosemary from tablelands) or “candeia-de-queimar” (burning candle), names that refer to its strong aromatic properties, and is popularly used in northeastern Brazil to treat respiratory and skin disorders, particularly wounds, irritations and infections [31,32,33].

This species has attracted increasing scientific interest due to its pharmacochemical potential, with reports describing antinociceptive, antimicrobial and cytotoxic activities [34,35,36]. These biological effects are largely associated with its essential oil, which commonly presents thymol- and carvacrol-rich chemotypes, supporting the ethnopharmacological importance of the species [37,38]. However, reports on its antifungal effects against dermatophytes are still limited [39].

Considering that dermatophytes primarily colonize keratinized tissues such as skin and nails, knowledge of how the essential oil of this traditionally used plant affects fungal growth, biofilm formation, and host cell responses remains limited. Therefore, the present study aimed to investigate the antifungal potential of *L. gracilis* essential oil, obtained from an access collected in northeastern Brazil, against dermatophytes and to evaluate its effects on biofilm formation and disruption, fungal morphology, and parameters associated with nail and skin infections, as well as its potential contribution to wound healing processes related to skin infections.

## 2. Results

### 2.1. Phytochemical Profile of Lippia gracilis Essential Oil

**Figure 1:**
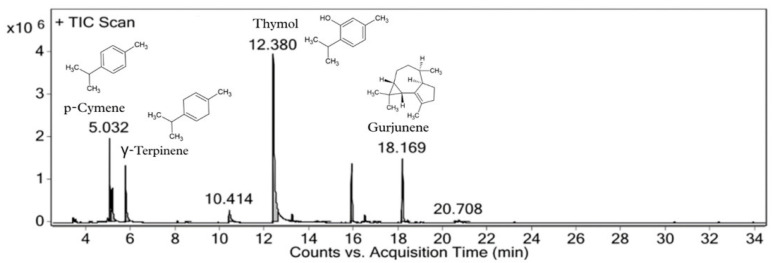
Total ion chromatogram (GC–MS) of the essential oil from *L. gracilis* fresh leaves, showing the main identified constituents: *p*-cymene, γ-terpinene, thymol, and gurjunene.

The essential oil obtained from fresh leaves of *L. gracilis* collected in Fortaleza, Ceará (Brazil) yielded 0.85% (*v*/*w*). GC–MS analysis revealed that the oil was predominantly composed of oxygenated monoterpenes (41.70%) and monoterpene hydrocarbons (29.14%) (Table 1). Thymol (37.52%) was the major constituent, followed by *p*-cymene (12.13%), gurjunene (10.95%) and γ-terpinene (9.29%) (Figure 1). The identified components accounted for 84% of the total oil composition, confirming the predominance of thymol and biogenetically related hydrocarbons (*p*-cymene and γ-terpinene).

### 2.2. Antifungal Activity of Lippia gracilis Essential Oil

#### 2.2.1. Minimal Inhibitory Concentration and Minimal Fungicidal Concentration of *L. gracilis* Essential Oil

*L. gracillis* essential oil exhibited important antifungal activity against dermatophyte strains, with MIC values ranging from 50 to 100 µg/mL and MFC values between 50 and 400 µg/mL (Table 2). Among the tested strains, *Epidermophyton floccosum*, *Microsporum canis*, *Microsporum gypseum*, *Trichophyton mentagrophytes*, and *Trichophyton verrucosum* were the most susceptible, with MIC values as low as 50 µg/mL. For *E. floccosum*, the essential oil exhibited a fungicidal effect, showing identical MIC and MFC values (50 µg/mL).

In contrast, the activity against *Candida* strains was lower, with MIC values ≥ 400 µg/mL and MFC values ≥ 800 µg/mL for most strains. Only *Candida tropicalis* showed comparatively higher susceptibility, with a MIC of 400 µg/mL. These findings illustrate a difference in susceptibility to *L. gracillis* essential oil between filamentous dermatophytes and yeasts under the tested conditions.

#### 2.2.2. Morphological Alterations Induced in Dermatophytes by *L. gracilis* Essential Oil

**Figure 2:**
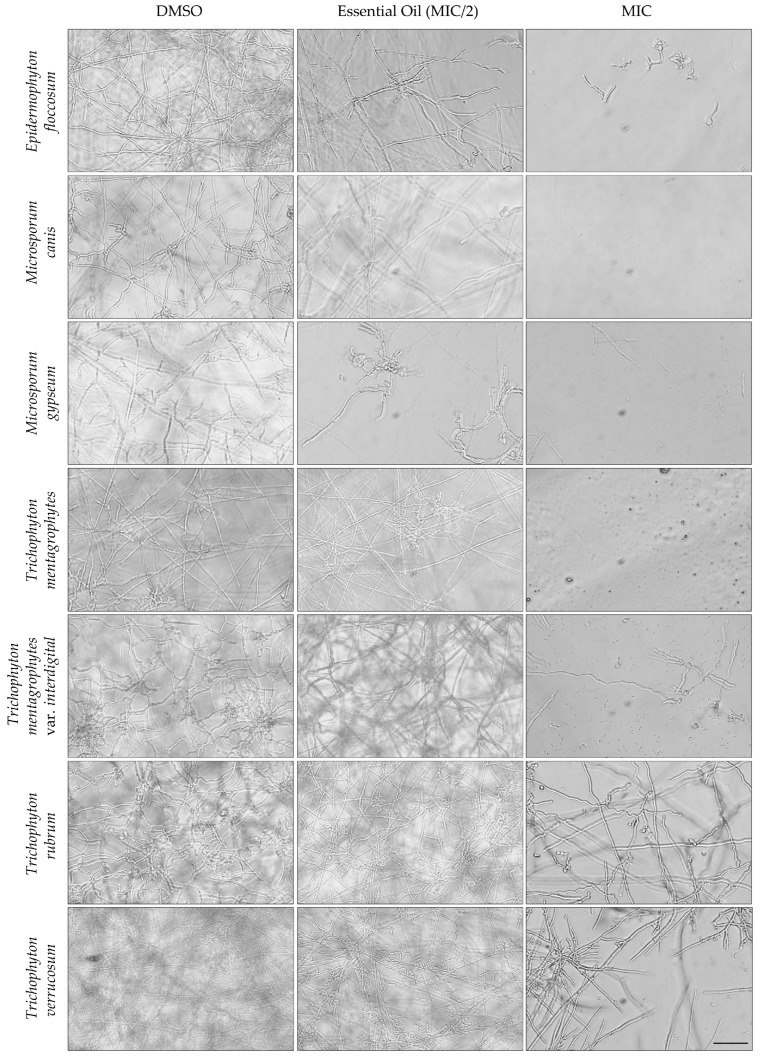
Morphological alterations in dermatophyte fungi exposed to *L. gracilis* essential oil at subinhibitory (MIC/2) and inhibitory concentrations (MIC).

Microscopic analysis of fungal morphology was performed using an optical microscopy at 40× magnification (Figure 2). The control samples (DMSO, without essential oil) displayed typical fungal architecture, with long, continuous, and well-organized hyphae forming dense and structured mycelial networks. In cultures exposed to the essential oil at sub-inhibitory concentrations (MIC/2), initial signs of hyphal disorganization were evident, characterized by irregular contours, wavy and thinner filaments, and a reduction in mycelial density. At the inhibitory concentration (MIC), as expected, the morphological damage became more pronounced, with disrupted and fragmented hyphae, formation of rounded or collapsed structures suggestive of cellular degeneration, and significant impaired mycelial growth. These alterations indicate a concentration-dependent antifungal effect, consistent with the loss of structural integrity under increasing exposure to the essential oil.

#### 2.2.3. Effect of *L. gracilis* Essential Oil on Immature Biofilms

**Figure 3:**
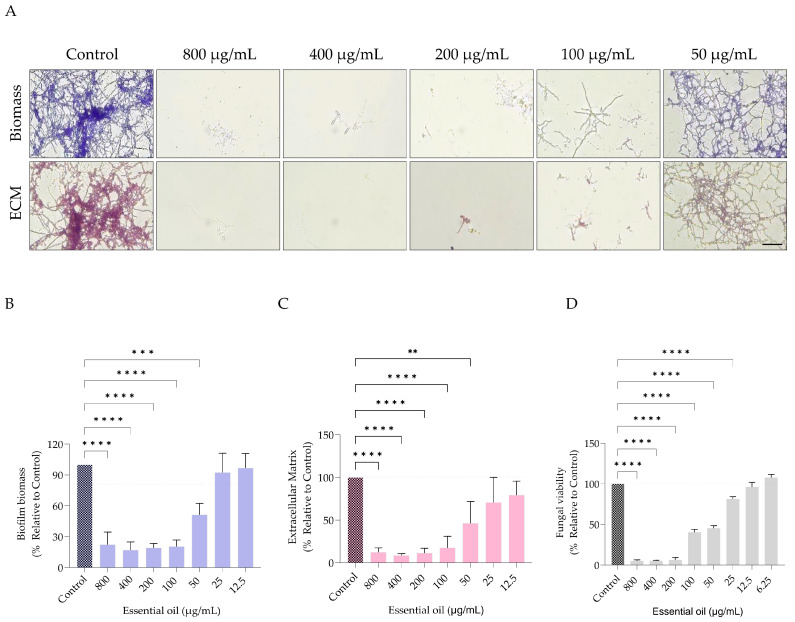
Immature biofilms of *Epidermophyton floccosum* untreated (control) or treated with essential oils from *L. gracilis*. Representative microscopy images of biofilm biomass stained with crystal violet and biofilm extracellular matrix stained with safranin (**A**). Quantification of biofilm biomass (**B**), biofilm extracellular matrix (**C**) and biofilm viability following XTT assay (**D**). Results are expressed as mean ± SEM values from at least three independent experiments. Statistical analysis was performed using one-way ANOVA followed by Dunnett’s multiple comparisons test to compare each treatment group with the control. Statistical significance was defined as ** *p* < 0.01, *** *p* < 0.001 and **** *p* < 0.0001, in comparison to control. The dashed horizontal line indicates the untreated control, normalized to 100%. Scale bar = 50 μm.

*L. gracilis* essential oil compromised the development of immature biofilms of *E. floccosum* in a concentration-dependent manner, reducing biomass, extracellular matrix deposition, and cell viability (Figure 3). Biomass quantification (Figure 3B) demonstrated that concentrations at and above the MIC (50 µg/mL) were highly effective, leading to substantial reductions in biomass (approximately 70–90%), compared with the untreated control. At sub-MIC levels (25 µg/mL), a moderate decrease was still observed, whereas lower concentrations (≤12.5 µg/mL) showed minimal inhibition, approaching control values. A similar effect was observed for extracellular matrix deposition (Figure 3C), with concentrations ≥ MIC promoting reductions (approximately 80–95%), while sub-MICs were less effective. Cell viability (Figure 3D) was also reduced. Even at low concentrations (notably at 50 µg/mL), viability declined by approximately 60–80% compared to the control, demonstrating pronounced antifungal activity under these conditions.

Overall, *L. gracillis* essential oil exhibited inhibitory activity against immature biofilms of *E. floccosum*, with reductions generally surpassing 50% at concentrations ≥50 µg/mL. These findings emphasize its interference with early biofilm establishment and structural integrity.

#### 2.2.4. Effect of *L. gracilis* Essential Oil on Mature Biofilms

**Figure 4:**
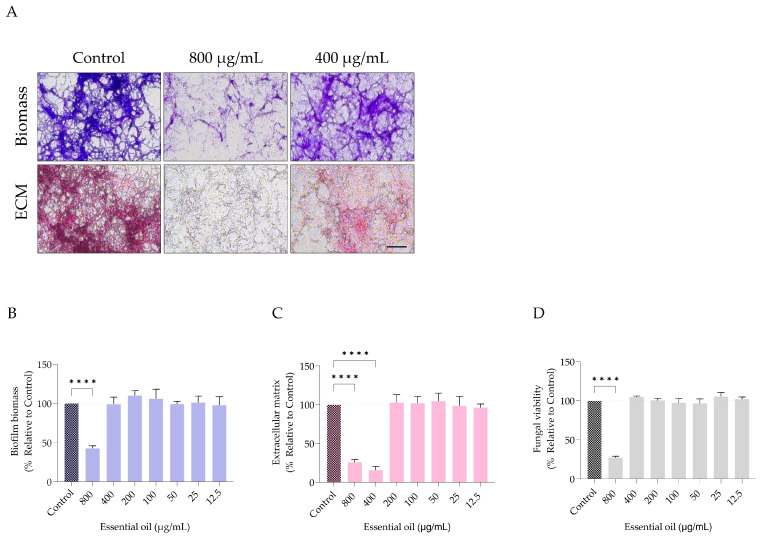
Mature biofilms of *Epidermophyton floccosum* untreated (control) or treated with essential oils from *L. gracilis*. Representative microscopy images of biofilm biomass stained with crystal violet and biofilm extracellular matrix stained with safranin (**A**). Quantification of biofilm biomass (**B**), biofilm extracellular matrix (**C**), and biofilm viability following XTT assay (**D**). Results are expressed as mean ± SEM values from at least three independent experiments. Statistical analysis was performed using one-way ANOVA followed by Dunnett’s multiple comparisons test to compare each treatment group with the control. Statistical significance was defined as **** *p* < 0.0001, in comparison to control. The dashed horizontal line indicates the untreated control, normalized to 100%. Scale bar = 50 μm.

*L. gracilis* essential oil was also able to reduce biofilm biomass, extracellular matrix, and cell viability in mature *E. floccosum* biofilms at the highest concentration tested (800 µg/mL) (Figure 4). Interestingly a reduction in extracellular matrix deposition was also observed at 400 µg/mL, with an inhibition around 80% in comparison to the control (Figure 4C).

#### 2.2.5. Preventive Potential of *L. gracilis* Essential Oil in an Ex Vivo Model of *Trichophyton rubrum*-Induced Onychomycosis

**Figure 5:**
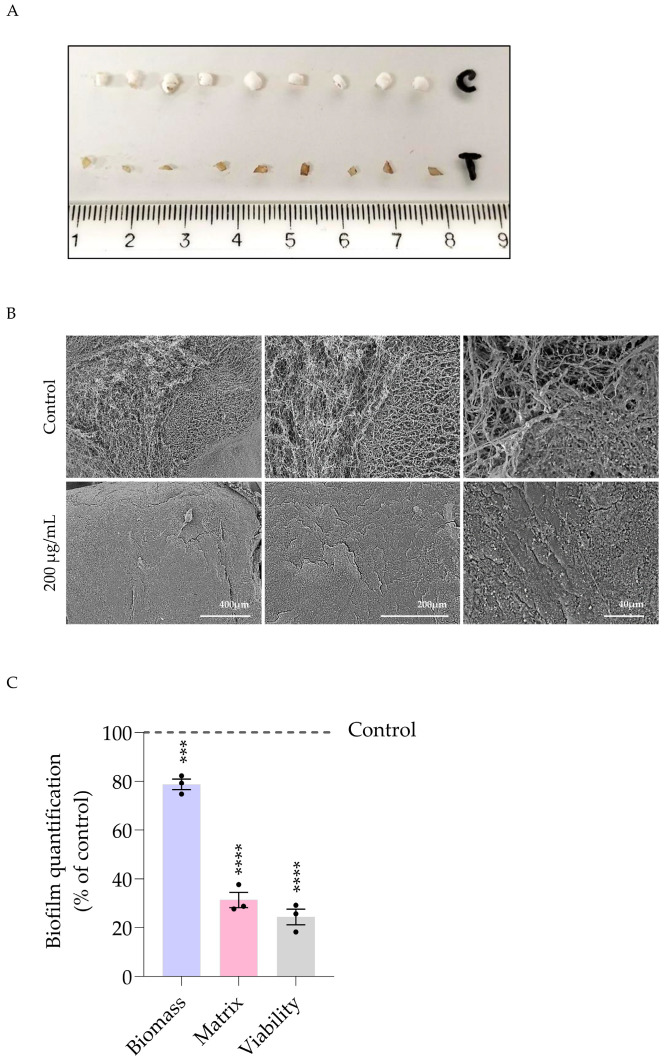
Preventive effect of *L. gracilis* essential oil (200 µg/mL) against *Trichophyton rubrum* biofilm formation in an ex vivo nail model. (**A**) Macroscopic analysis of infected nail fragments after treatment, where “C” represents the untreated control group and “T” represents the group treated preventively with *L. gracilis* essential oil. (**B**) Scanning electron microscopy (SEM) images showing surface morphology of *T. rubrum* biofilm on nail fragments. (**C**) Quantification of biofilm biomass, extracellular matrix, and cell viability following treatment with *L. gracilis* essential oil. Results are expressed as mean ± SEM values from at least three independent experiments. Statistical analysis was performed using one-way ANOVA followed by Dunnett’s multiple comparisons test to compare each treatment group against the control. Statistical significance is indicated as *** *p* < 0.001 and **** *p* < 0.0001, in comparison to the control.

Macroscopic analysis of the infected nails showed that the untreated control fragments exhibited a whitish, cotton-like appearance, consistent with dermatophyte colonization and extensive fungal growth (Figure 5A). In contrast, the fragments treated with *L. gracilis* essential oil (200 µg/mL) appeared more preserved and closer to the natural expected nail aspect. These observations suggest that the treatment reduced the visible impact of fungal colonization on the nail surface in the preventive assay. Scanning electron microscopy (SEM) analysis supported these findings, as control samples displayed a dense and organized filamentous network covering the nail surface, compatible with active fungal growth and biofilm formation (Figure 5B). By comparison, treated samples, in the preventive assay, showed a marked reduction in filamentous structures, a more disrupted surface pattern, and partial preservation of the nail architecture (Figure 5B). Although some surface irregularities were observed, the treated fragments exhibited a substantially less colonized appearance than the control, indicating that *L. gracilis* essential oil interfered with *T. rubrum* biofilm development in the ex vivo onychomycosis model.

Corroborating these findings, the quantitative data presented in Figure 5C demonstrate that *L. gracillis* essential oil reduced biofilm biomass, matrix production, and fungal viability relative to the untreated control. Together, these results indicate that the treatment effectively impaired both the structural establishment and the persistence of *T. rubrum* biofilm.

#### 2.2.6. Therapeutic Efficacy of *L. gracilis* Essential Oil in an Ex Vivo Model of *Trichophyton rubrum*-Induced Onychomycosis

**Figure 6:**
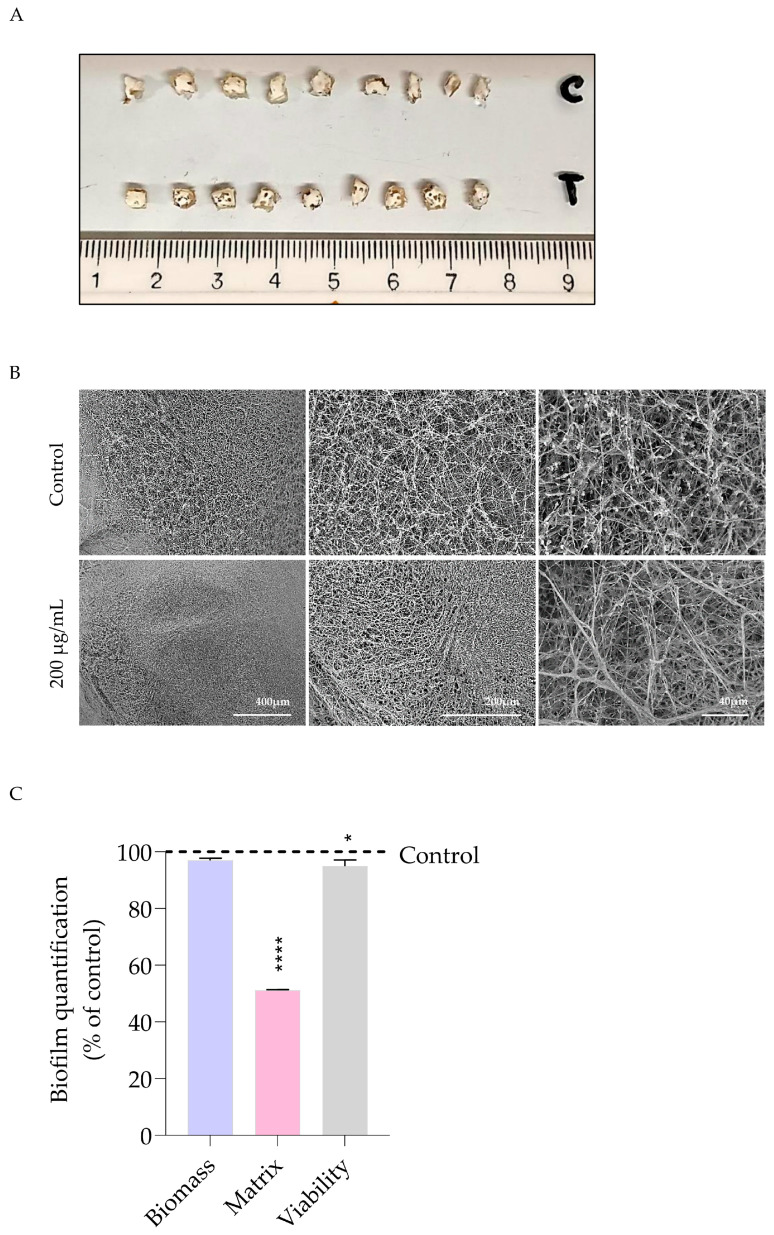
Therapeutic effect of *L. gracilis* essential oil (200 µg/mL) against pre-established *Trichophyton rubrum* biofilms in an ex vivo nail model. (**A**) Macroscopic analysis of infected nail fragments after treatment, where “C” represents the untreated control group and “T” represents the group treated with *L. gracilis* essential oil. (**B**) Scanning electron microscopy (SEM) images showing surface morphology of pre-established *T. rubrum* biofilm on nail fragments. (**C**) Quantification of biofilm biomass, extracellular matrix, and cell viability following treatment with *L. gracilis* essential oil. Results are expressed as mean ± SEM values from at least three independent experiments. Statistical analysis was performed using one-way ANOVA followed by Dunnett’s multiple comparisons test to compare each treatment group with the control. Statistical significance is indicated as * *p* < 0.05 and **** *p* < 0.0001, in comparison to the control.

In the post-infection treatment assay, the macroscopic appearance of the nail fragments indicated a low effect of *L. gracilis* essential oil (Figure 6A). Compared with the untreated control, the treated samples showed little reduction in the whitish and cotton-like fungal overgrowth, with residual surface alterations and evident signs of colonization. SEM observations (Figure 6B) confirmed the limited effect of the essential oil in the post-infection follow-up assay. At lower magnification (×250), control samples exhibited a dense, well-developed filamentous network covering the nail surface, consistent with fungal colonization and biofilm maturation. In treated samples, the fungal structures appeared slightly less compact and more disorganized, with partial reduction in hyphal density and surface coverage. Nevertheless, substantial filamentous growth remained, indicating incomplete disruption of the established biofilm. At higher magnification (×2000), globular and aggregated structures were observed interspersed within the filamentous network, which may correspond to extracellular matrix-associated material. In the control samples, these structures were more abundant and contributed to a dense, compact surface layer, whereas the treated samples displayed a less organized network with reduced coverage. This suggests that the essential oil affected the architecture of the fungal biofilm, although its inhibitory effect was limited (Figure 6C).

Quantitative analysis (Figure 6C) supported the microscopic observations. While no significant reduction in total biomass was detected compared to the control, a marked decrease in biofilm matrix was observed, indicating a significant disruption of the extracellular polymeric components. In contrast, fungal viability was only slightly affected, remaining close to control levels. These results suggest that the essential oil interferes with the structural integrity of the biofilm matrix, although it has limited impact on fungal survival within pre-established biofilms.

### 2.3. Wound Healing Potential of Lippia gracilis Essential Oil

#### 2.3.1. Safety Profile of *L. gracilis* Essential Oil on Skin Cells

**Figure 7:**
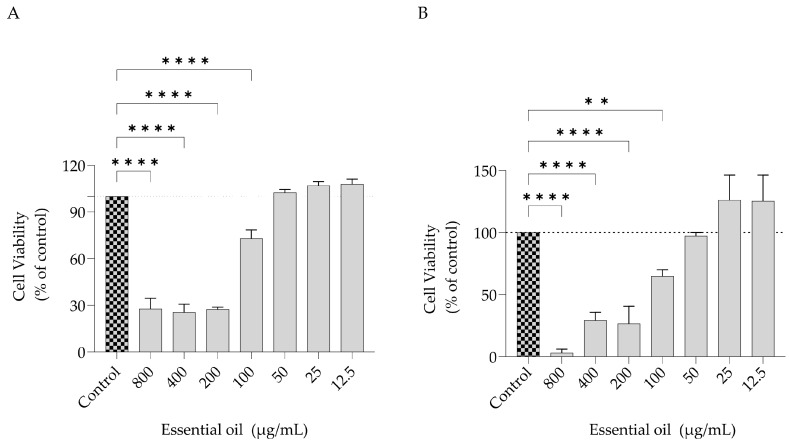
Dose-dependent effects of *L. gracilis* essential oil (800–12.5 µg/mL) on the viability of 3T3 fibroblasts (**A**) and HaCaT keratinocytes (**B**), determined by the resazurin assay. Results are expressed as mean ± SEM values from at least three independent experiments. Statistical analysis was performed using one-way ANOVA followed by Dunnett’s multiple comparisons test to compare each treatment group against the untreated control. Statistical significance is indicated as ** *p* < 0.01 and **** *p* < 0.0001, in comparison to control. The dashed horizontal line indicates the untreated control, normalized to 100% cell viability.

Cell viability was assessed using the resazurin assay following exposure of 3T3 fibroblasts and HaCaT human keratinocytes to *L. gracilis* essential oil (800–12.5 µg/mL) (Figure 7). Fibroblasts (Figure 7A) maintained viability at concentrations ≤50 µg/mL, indicating no detectable cytotoxicity within this range, while reductions were observed only at higher concentrations (800–100 µg/mL) in a dose-dependent manner. HaCaT cells (Figure 7B) showed a comparable pattern, with viability preserved at ≤50 µg/mL and cytotoxic effects occurring predominantly at elevated concentrations.

#### 2.3.2. Evaluation of Cell Migration

**Figure 8:**
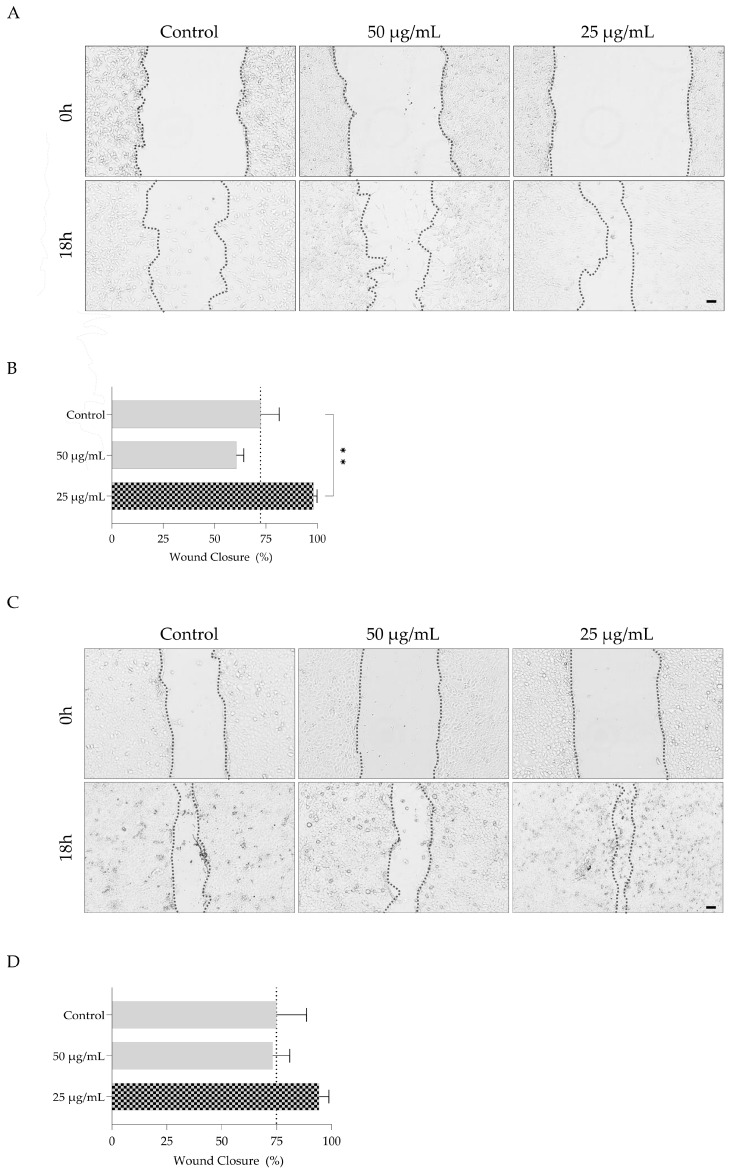
Effects of *L. gracilis* essential oil (50–25 µg/mL) on cell migration evaluated by the wound healing assay. Representative microscopy images acquired at 0 h and 18 h after scratch induction and treatment with *L. gracilis* essential oil in fibroblasts (**A**) and keratinocytes (**C**); the wound area is outlined by dashed lines. Quantification of cell migration based on wound area closure after 18 h of treatment in fibroblasts (**B**) and keratinocytes (**D**). Data are expressed as mean ± SEM of at least three independent experiments and are presented relative to the control −100% migration. Statistical analysis was performed using one-way ANOVA followed by Dunnett’s multiple comparisons test: ** *p* < 0.01, compared to control.

*L. gracilis* essential oil influenced cell migration in a concentration-dependent manner in both fibroblasts and keratinocytes (Figure 8). In fibroblasts (Figure 8A,B), the control group exhibited wound closure after 18 h. Treatment with *L. gracillis* essential oil at 50 µg/mL impaired cell migration, indicating a marked inhibitory effect at higher concentrations. In contrast, exposure to 25 µg/mL of the essential oil enhanced fibroblast migration compared with the control, suggesting a stimulatory effect at lower concentrations.

A comparable pattern was observed in HaCaT cells (Figure 8C,D). Treatment with 50 µg/mL of *L. gracilis* essential oil resulted in a noticeable reduction in wound closure relative to the control; however, this effect did not reach statistical significance under the tested conditions. Similarly, treatment at 25 µg/mL promoted cell migration, although no statistical significance was attained.

## 3. Discussion

Dermatophytoses are neglected infections with a substantial global burden, affecting millions of people annually, yet they receive less research and development attention compared to other fungal diseases and are absent from the WHO’s priority pathogen list [40]. Although terbinafine has demonstrated historical efficacy as first-line therapy against dermatophytosis, emerging resistance, now undermines treatment outcomes, leading to high recidivism cases [41]. Over 30 species, encompassing anthropophilic, zoophilic, and geophilic strains, require optimized therapeutic regimens, rapid antifungal susceptibility testing, and clinical trials evaluating alternatives alongside infection control measures [42], thereby illustrating the therapeutic potential of essential oils like *L. gracilis* in addressing this gap.

The essential oil from fresh leaves of *L. gracilis* exhibited a thymol-dominant profile (37.52%), accompanied by *p*-cymene (12.13%), γ-terpinene (9.29%), and gurjunene (10.95%). This chemical composition aligns with the thymol-rich chemotype commonly reported for *L. gracilis* specimens from northeastern Brazil, particularly in the Ceará State [34,43,44]. While registered accessions from the northeastern Brazilian region exhibit chemotype variability (thymol- or carvacrol-dominant) [45,46,47], this specific Ceará access is thymol-rich. Importantly, historical analyses of the same accession performed more than three decades ago by Lemos et al. [48] also demonstrated a similar phytochemical profile, characterized by thymol (30.6%) and *p*-cymene (10.7%), supporting the long-term chemical stability of this germplasm accession, likely associated with the maintenance of its genetic background through asexual reproduction [49]. Such intraspecific diversity emphasizes the importance of chemical standardization, especially for germplasm banks that supply matrix plants to Brazil’s public phytotherapy programs, including the ‘*Farmácias Vivas*’ (Living Pharmacies) network where consistent phytochemical profiles ensure therapeutic efficacy, safety, and reproducibility in large-scale production of herbal medicines for the SUS (Sistema Único de Saúde). Previous studies [50,51,52,53,54,55,56] have demonstrated the long-term maintenance of chemical characteristics in species from the Medicinal Plants Garden Professor Francisco José de Abreu Matos germplasm bank over decades, further supporting the reliability of this collection for sustainable phytotherapeutic supply chains.

In terms of antifungal activity, *L. gracilis* essential oil exhibited potent antifungal activity against dermatophyte strains, with MIC values ranging from 50 to 100 µg/mL and MFC values from 50 to 400 µg/mL (Table 2). Previous studies on *L. alba* chemotypes have also demonstrated antidermatophytic effects, with linalool-rich essential oils showing MIC values of 156 and 312 µg/mL against *E. floccosum* and *M. gypseum* [57] and citral-chemotype essential oils achieving MIC values of 125 µg/mL against *T. mentagrophytes* [58]. Herein, *L. gracilis* thymol-rich essential oil showed higher potency for these strains, demonstrating its advantage over congeneric species.

Considering that *L. gracilis* essential oil is rich in thymol, it is reasonable to speculate that the antifungal activity observed in this study may be related, at least in part, to the presence of this compound, which could contribute to the overall bioactivity of the oil. Indeed, a previous study showed comparable results for isolated thymol with a MIC value of 100 µg/mL against *M. canis* and MIC values ranging from 50 to 100 µg/mL against *T. mentagrophytes* [59]. In addition, previous studies from our group also showed effective antidermatophytic activity for thymol with MIC values ranging from 0.08 to 0.32 µL/mL [60], comparable to that observed for the whole essential oil in the present study (50–100 µg/mL). The antifungal potential of thymol has been correlated with multiple antifungal mechanisms, particularly inhibition or disruption of ergosterol biosynthesis [61,62], induction of lipid peroxidation [63], suppression of plasma membrane H^+^-ATPase activity [64], and direct damage to the plasma membrane integrity [65], thereby justifying its potent antifungal effect. Nevertheless, this compound has shown cytotoxicity toward skin cells, with IC_50_ values of 57.0 µg/mL for MCF10A mammary epithelial cells and of 82.0 µg/mL for HdFn neonatal fibroblasts [66]. In contrast, *L. gracilis* essential oil was shown in the present study to be safe for fibroblasts and keratinocytes at the MIC against most of the dermatophytes tested (50 µg/mL), suggesting a more favorable therapeutic window for topical applications.

In comparison with fluconazole, a widely used standard antifungal agent, the essential oil showed comparable activity against several dermatophytes, with MIC values of 50–100 µg/mL, which lie within the reported range for fluconazole (16–128 µg/mL) [67], suggesting a similar level of in vitro efficacy.

Our study addresses a gap in the literature by demonstrating the antibiofilm efficacy of a thymol-rich natural extract against dermatophyte biofilms, for which evidence on thymol-dominant phytocomplexes remains limited. The use of the essential oil rather than an isolated compound may reduce resistance development and provide broader-spectrum activity due to its complex chemical composition, which can involve multiple bioactive constituents acting on different cellular targets and potentially exert synergistic antifungal effects [68,69]. Based on this rationale, we evaluated the effect of the essential oil on biofilm formation and development. Previous studies have demonstrated that *E. floccosum* is capable of forming biofilms in vitro, supporting its use as a relevant organism for antibiofilm evaluation among dermatophytes [9,70]. This fungus was selected as the biological model due to its high clinical and epidemiological relevance, as it is a well-recognized causative agent of dermatophytic skin infections, particularly tinea cruris, tinea pedis, and tinea corporis [71]. *E. floccosum* is the only species currently classified within the genus *Epidermophyton* [72] and is phylogenetically distinct from the genera *Microsporum* and *Trichophyton* [73], which represent the other major groups of dermatophytes. This clear taxonomic and phylogenetic distinction supports its use as a well-defined and standardized model organism, facilitating interpretation of antifungal activity without the complexity of multiple species within the same genus.

Regarding antibiofilm properties, *L. gracilis* essential oil exhibited superior activity against immature biofilms compared to mature ones, significantly reducing biomass, extracellular matrix, and cell viability at concentrations as low as 100 µg/mL (Figure 3), reflecting a greater vulnerability of early-stage communities prior to full development. In mature biofilms (Figure 4), significant reductions occurred primarily at 800 µg/mL for biomass and fungal viability, and at 400 µg/mL for matrix deposition, consistent with the tolerance conferred by a dense extracellular matrix, which may hinder compound penetration. These results are consistent with plants essential oils’ more effective interference in early adhesion and extracellular matrix production stages rather than overcoming the diffusion barrier of established biofilms [74].

Once again, thymol, the major compound of *L. gracilis* essential oil is well known for its antifungal activity against biofilms. It inhibits biofilm formation by disrupting initial adhesion, cell aggregation, and biofilm-associated gene expression, as well as by compromising membrane integrity and inducing oxidative stress, thereby impairing biofilm development and fungal survival [75,76,77]. However, its reduced efficacy against mature biofilms may be attributed to the protective role of the extracellular matrix, which limits compound penetration and restricts access to embedded cells. This mechanism might explain the superior activity of *L. gracilis* essential oil against immature biofilms (100 µg/mL) compared to established structures, which require higher concentrations (400–800 µg/mL).

Since dermatophyte biofilm formation on keratinized tissues contributes to persistent infection, reduced antifungal susceptibility, and therapeutic failure in onychomycosis, we evaluated the effect of the oil in a clinically relevant ex vivo model. *Trichophyton rubrum* was selected for the nail model due to its predominance as the main etiological agent of onychomycosis worldwide [78], thereby ensuring the clinical relevance of the experimental system. This species forms biofilms on human nail fragments, exhibiting greater biomass and extracellular matrix accumulation ex vivo compared to polystyrene microplates [79,80]. In this sense, ex vivo models for dermatophyte growth employ natural substrates to recapitulate in vivo nutritional and environmental conditions, unlike artificial in vitro media. This approach yields biofilms with preserved extracellular matrix and complex architecture, conferring greater antifungal tolerance [81]. Accordingly, our human nail model employed concentrations two times higher than the MIC to account for penetration barriers in dead keratinized tissue, thereby providing evidence toward potential translation to clinical onychomycosis. Our results demonstrate that *L. gracilis* essential oil in ex vivo experiments followed the trend observed in vitro for biofilm assays, with the preventive assay (analogous to immature biofilm formation) proving to be more effective than the post-infection treatment assay (analogous to mature biofilm disruption). This pattern underscores the oil’s superior prophylactic potential against early biofilm establishment in keratinized human nails over 10-day short-term experiments, suggesting its preferential application in cosmeceutical formulations such as protective nail varnishes and polishes for onychomycosis prevention and management.

The extracellular matrix of dermatophyte biofilms acts as a physical barrier that limits the penetration of conventional antifungals. This effect is further enhanced by metabolic cooperation and the upregulation of resistance genes, rendering biofilms significantly more tolerant than planktonic cells [7,82]. At 400 µg/mL, *L. gracilis* essential oil exhibited activity against resistant nail biofilms, likely through targeting biofilm extracellular matrix. These findings support the cautious topical application of this oil in onychomycosis, where the keratinized nail limits systemic and skin exposure, while enabling activity against biofilm-associated resistance mechanisms.

The safety profile of *L. gracilis* essential oil indicates a concentration-dependent therapeutic window in relation to its antifungal activity. While it was non-toxic at ≤50 µg/mL to fibroblasts and keratinocytes, this MIC-equivalent concentration did not promote wound closure. In contrast, 25 µg/mL significantly enhanced fibroblast migration (*p* < 0.01), indicating a proliferative effect at sub-antifungal concentrations. Although the increase in keratinocyte migration at 25 µg/mL was not statistically significant, a consistent upward trend was observed. Importantly, these findings also suggest a degree of overlap between antifungal and host–cell active concentrations. In particular, concentrations close to the MIC appear to be less favorable for cell migration, indicating a relatively narrow therapeutic window and warranting cautious interpretation of potential topical applications. This apparent concentration-dependent divergence is in line with reports for thymol-rich essential oils, which may promote wound healing through modulation of inflammation, oxidative stress, re-epithelialization, angiogenesis, granulation tissue formation, and collagen deposition, but also exhibit cytotoxic effects at higher concentrations, requiring careful dose optimization [83]. Taken together, these findings suggest that the essential oil has different effects depending on the concentration used. In particular, increased cell migration was seen at lower, sub-antifungal concentrations, while this effect was not observed at concentrations close to the MIC. This is relevant for wound healing, since fibroblast migration plays a central role in tissue repair and regeneration, and it also highlights the importance of careful dose optimization for potential topical applications.

## 4. Materials and Methods

### 4.1. Plant Material and Essential Oil Extraction

The plant material was sourced from the active germplasm bank of the Professor Francisco José de Abreu Matos Medicinal Plants Garden, located at the Universidade Federal do Ceará, Pici Campus, Fortaleza, Ceará, Brazil (3°44′43.89″ S, 38°34′37.67″ W). Aerial parts were collected using disinfected professional pruning shears (Tramontina^®^, Carlos Barbosa, Brazil), which had been disinfected beforehand, at 09:00 a.m. during the dry season in September 2024. A voucher specimen (HPL 8785) was deposited in the Herbarium of Jardim Botânico Plantarum (HPL), Nova Odessa, São Paulo, Brazil.

The access to Brazilian genetic heritage was registered in the National System for the Management of Genetic Heritage and Associated Traditional Knowledge (SisGen), in compliance with Law No. 13.123/2015 and its regulations, under registration code A8C5BBF, for research and technological development purposes.

The material was briefly processed by removing insects, stems, and flowers, with only fresh leaves subjected to steam distillation for 2 h, as described by Craveiro et al. [84] with adaptations made by Castro et al. [50]. The essential oil obtained was treated with anhydrous sodium sulfate to remove residual moisture and aliquoted into amber glass vials stored at 4 °C until chemical analyses and further assays were performed.

### 4.2. Phytochemical Profiling of the Essential Oil

Gas chromatography–mass spectrometry (GC–MS) profile was obtained using an Agilent 7890B gas chromatograph (Agilent Technologies, Santa Clara, CA, USA) coupled to a 5977A quadrupole-type mass spectrometer (Agilent Technologies, Santa Clara, CA, USA). Separation was carried out on an HP-5 MS capillary column (30 m × 0.25 mm × 0.25 μm Agilent Technologies, Santa Clara, CA, USA). The injector, detector, and transfer line were maintained at 250 °C, 150 °C, and 280 °C, respectively. The oven temperature was initially set to 70 °C, raised at 4 °C/min to 180 °C, and then at 10 °C/min up to 250 °C, with a total run time of approximately 34 min. Mass spectra data were acquired using the MassHunter GC/MS Data Acquisition software (version B.07.02.1938, Agilent Technologies, Santa Clara, CA, USA). Compound identification was based on comparison of the obtained spectra with those in the NIST mass spectral library. Retention indices were calculated using a homologous series of n-alkanes (C7–C30) from Sigma-Aldrich (St. Louis, MO, USA) under the same chromatographic conditions, and the experimental retention indices were compared with literature data [85,86].

### 4.3. Antifungal Activity

#### 4.3.1. Fungal Strains and Culture Conditions

The antifungal activity of *L. gracilis* essential oil was evaluated against several pathogenic fungal strains, including dermatophytes and yeasts. The panel of dermatophytes included three clinical isolates obtained from nail and skin infections, namely *Epidermophyton floccosum* (FF9), *Microsporum canis* (FF1), and *Trichophyton mentagrophytes* (FF7). Four dermatophyte reference strains were also tested: *M. gypseum* CECT 2908, *T. mentagrophytes* var. *interdigitale* CECT 2958, *T. rubrum* CECT 2794, and *T. verrucosum* CECT 2992 (Valencia, Spain). The yeast group comprised two clinical *Candida* isolates obtained from recurrent cases of vulvovaginal and oral candidiasis (*C. krusei* H9 and *C. guilliermondii* MAT23), as well as three reference *Candida* strains: *C. albicans* ATCC 10231, *C. parapsilosis* ATCC 90018, and *C. tropicalis* ATCC 13803 (Manassas, VA, USA). All fungal strains belong to the microorganism collection of the Pharmacognosy Laboratory, Faculty of Pharmacy, University of Coimbra (Coimbra, Portugal).

Before each experiment, the cultures were subcultured on Sabouraud Dextrose Agar (SDA; Oxoid, Thermo Fisher Scientific, Waltham, MA, USA) to ensure viability. *Candida* strains were incubated at 35 °C for 48 h in a dedicated yeast incubator, while dermatophyte strains were incubated at 30 °C for approximately 5 days in a separate incubator. Fungal inocula were prepared in 0.9% NaCl and adjusted to a 0.5 McFarland standard (1–5 × 10^6^ CFU/mL) using a DEN-1 turbidimeter (BioSan, Riga, Latvia).

#### 4.3.2. Determination of the Minimum Inhibitory and Minimum Fungicidal Concentrations

The broth microdilution method was employed to determine the minimum inhibitory concentrations (MICs) and the minimum fungicidal concentrations (MFCs) of *L. gracilis* essential oil, following EUCAST reference protocols E.DEF 7.4 (yeasts) [87] and E.DEF 11.0 (filamentous fungi) [88]. Briefly, a stock solution of *L. gracilis* essential oil was initially prepared in DMSO at 100 mg/mL and serially diluted in RPMI 1640 medium (Sigma-Aldrich, St. Louis, MO, USA) across a concentration range of 6.25 to 800 µg/mL. Positive controls consisted of DMSO at the highest concentration used (≤0.7% *v*/*v*) to ensure that the solvent did not affect fungal growth, while negative controls contained 0.9% NaCl. The MIC was determined as the lowest concentration showing no macroscopically visible fungal growth following an incubation period of 48 h for *Candida* spp. or 5 days for dermatophytes. For MFC determination, 20 µL aliquots from negative wells were subcultured onto SDA plates and incubated for equivalent duration (48 h for *Candida* or 5 days for dermatophytes). The lowest concentration showing no growth corresponded to the minimum fungicidal concentration (MFC).

#### 4.3.3. Microscopic Evaluation of Fungal Morphology

Photomicrographs were acquired using an inverted light microscope (VisiScope IT417PH, VWR International, Radnor, PA, USA) at 40× magnification. Morphological changes were assessed qualitatively by direct observation of fungal structures in the microplate wells, comparing treated groups at MIC/2 and MIC values with that of the control (DMSO), with emphasis on hyphal organization, structural integrity, and mycelial density.

### 4.4. Antibiofilm Activity Against Epidermophyton floccosum

#### 4.4.1. Culture Conditions

The antibiofilm activity of *L. gracilis* essential oil against *E. floccosum* was assessed on both immature and mature biofilms, following the methodology described by Ali et al. [89], with minor modifications previously optimized by Alves-Silva et al. [90]. In the first assay, following an initial fungal adhesion period of 3 h, adherent fungal cells were co-incubated with *L. gracilis* essential oil (6.25–800 µg/mL) for an additional 72 h period. In the mature biofilm assay, pre-formed biofilms (3 h adhesion followed by 72 h of incubation in culture medium) were exposed to the essential oil (6.25–800 µg/mL) for an additional 24 h period. Both assays were conducted in 96-well polystyrene microplates and cultures were maintained at 37 °C.

#### 4.4.2. Quantification of Biofilm Biomass, Extracellular Matrix Deposition and Fungal Viability

Biofilm biomass was determined by crystal violet staining following the procedure optimized by Castelo-Branco et al. [81], with adaptations. After staining, excess dye was removed thorough washing, and bound crystal violet was solubilized using an acetic acid solution (33% *v*/*v*). Absorbance was recorded at 620 nm using a Multiskan™ FC microplate spectrophotometer (Thermo Fisher Scientific, Waltham, MA, USA).

Extracellular matrix (ECM) deposition was quantified using safranin red, as described by Costa-Orlandi et al. [91]. The dye specifically binds to ECM components, and absorbance was measured at 520 nm using the Multiskan™ FC microplate spectrophotometer.

Biofilm metabolic activity was evaluated through the XTT reduction assay following the methodology established by Alves et al. [90]. The reduction in XTT by metabolically active fungal cells produces a colorimetric signal proportional to biofilm viability. Absorbance was quantified at 490 nm using the Multiskan™ FC microplate spectrophotometer.

### 4.5. Ex Vivo Nail Infection Model of Thrichophytum rubrum

#### 4.5.1. Infection Model

The ex vivo nail infection model was performed following the protocol described by Brown et al. [92] with adaptations. Distal nail clippings were obtained from healthy adult volunteers during routine personal hygiene procedures. Samples were confirmed to be clinically healthy, free from visible signs of infection, dystrophy, or damage, and excluded if containing nail polish, artificial extensions, or cosmetic coatings. Clippings were placed on aluminum foil and autoclaved at 121 °C for 20 min. Sterile fragments (approximately 0.2 cm^2^) were then aseptically cut using sterile instruments.

Infection induction was performed by preparing a *T. rubrum* suspension adjusted to a 0.5 McFarland standard. 20 µL of the standardized inoculum was evenly spread onto Yeast Nitrogen Base (YNB) agar plates. Nail fragments were immediately positioned directly onto the inoculated surface, ensuring close contact and partial immersion to promote fungal adhesion and colonization. Inoculated nails were incubated at 25 °C for 10 days, with daily monitoring to confirm fungal growth and successful nail colonization.

#### 4.5.2. Essential Oil’s Preventive Potential Against Infection

The preventive activity of *L. gracilis* essential oil was evaluated as previously described by Brown et al. [92], with adaptations. Briefly, 5 μL of the essential oil at 200 ug/mL was applied to the surface of the nails, and the samples were allowed to dry at room temperature. Subsequently, treated nails were aseptically transferred onto YNB agar plates, previously inoculated with *T. rubrum*. The treatment was repeated once daily for 10 consecutive days. Untreated infected nail fragments served as negative controls. All samples were incubated at 25 °C throughout the treatment period and the experiments were performed in triplicate. At day 10, the following experiments were conducted: macroscopic and microscopic examination, biofilm biomass (crystal violet assay), extracellular matrix (ECM; safranin red assay), and viability (XTT assay) determinations.

#### 4.5.3. Essential Oil’s Post-Infection Therapeutic Effect

The post-infection therapeutic potential of *L. gracilis* essential oil against *T. rubrum*-infected human nail fragments was evaluated as previously reported by Brown et al. [92], with slight adaptations. Nail fragments were initially infected with a 20 µL inoculum of *T. rubrum* (0.5 McFarland, adjusted by densitometry) and incubated at 25 °C for 10 days, with daily monitoring of infection progression. The infected fragments were then aseptically transferred to fresh plates containing sterile Yeast Nitrogen Base (YNB) agar to initiate treatment. *L. gracilis* essential oil was prepared from a 100 mg/mL DMSO stock solution, diluted in 0.9% NaCl saline to 200 µg/mL, and applied directly to the nails (5 µL per fragment), once a day, for 10 consecutive days. Untreated infected nail fragments served as negative controls. All samples were incubated at 25 °C throughout the treatment period and the experiments were performed in triplicate. At day 20, the following experiments were conducted: macroscopic and microscopic examination, biofilm biomass (crystal violet assay), extracellular matrix (ECM; safranin red assay), and viability (XTT assay) determinations.

#### 4.5.4. Scanning Electron Microscopy (SEM) Analysis

Nail samples (control and infected) from both preventive and therapeutic assays were mounted on aluminum stubs using double-sided carbon adhesive tabs. Observations were carried out using a FlexSEM 1000 (Hitachi, Tokyo, Japan) scanning electron microscope operating in a variable-pressure mode and equipped with a cryo-stage. Images were acquired at 15 kV.

### 4.6. Cell Culture

Mouse fibroblasts (NIH/3T3, ATCC CRL-1658, Manassas, VA, USA) were cultured in Dulbecco’s Modified Eagle’s Medium (DMEM; 12800-017, Gibco, Thermo Fisher Scientific, Waltham, MA, USA) supplemented with 10% (*v*/*v*) heat-inactivated fetal bovine serum (FBS; Sigma-Aldrich, St. Louis, MO, USA), 1% (*v*/*v*) penicillin–streptomycin (Sigma-Aldrich, St. Louis, MO, USA), 3.7 g/L sodium bicarbonate (Sigma-Aldrich, St. Louis, MO, USA), and 25 mM glucose. The cells were maintained in 75 cm^2^ culture flasks at 37 °C in a humidified atmosphere of 5% CO_2_ and 95% air. Cells were subcultured at approximately 80% confluence using TrypLE Express (12605-028, Gibco, Thermo Fisher Scientific, Grand Island, NY, USA) to facilitate detachment. Cell morphology was routinely monitored under an inverted light microscope (VisiScope IT417PH, VWR International, Radnor, PA, USA).

Human immortalized keratinocytes (HaCaT; DKFZ, Heidelberg, Germany) were cultured in high-glucose DMEM supplemented with 2 mM L-glutamine (Sigma-Aldrich, St. Louis, MO, USA), 10% heat-inactivated FBS, and 1% (*v*/*v*) penicillin–streptomycin. Cultures were maintained in 75 cm^2^ flasks at 37 °C in a humidified atmosphere containing 5% CO_2_. Cells were subcultured at 80% confluence using TrypLE Express and cell morphology was monitored using an inverted light microscope.

### 4.7. Cell Viability

The effect of different concentrations of *L. gracilis* essential oil on the viability of fibroblasts and keratinocytes was evaluated using the resazurin reduction assay, as described by Alves-Silva et al. [93], with minor modifications. Fibroblasts were seeded in 48-well plates at 3.0 × 10^5^ cells/mL, whereas keratinocytes were seeded in 96-well plates at 1.5 × 10^5^ cells/mL. Essential oil concentrations were prepared from a stock solution in DMSO (100 mg/mL), followed by serial dilutions in the respective culture medium to reach the desired final concentrations. The final DMSO content in each well did not exceed 0.7% (*v*/*v*). After treatment, the medium was replaced with fresh medium containing resazurin (500 µM; 1:10), and the plates were incubated for 2 h at 37 °C. Absorbance was measured at 570 nm, with a reference wavelength of 620 nm, using an automated microplate reader (SLT, Salzburg, Austria). Cell viability was expressed as a percentage relative to the untreated control and calculated according to the equation:

$$\mathrm{Cell}\;\mathrm{viability}\;(\%)=\frac{{\mathrm{Abs}}_{\mathrm{Exp}}}{{\mathrm{Abs}}_{\mathrm{CT}}}\times 100$$

where ${\mathrm{Abs}}_{\mathrm{Exp}}$ is the absorbance difference (570–620 nm) of essential oil-treated cells, and ${\mathrm{Abs}}_{\mathrm{CT}}$ corresponds to the absorbance difference of control cells (without essential oil).

### 4.8. Cell Migration

Cell migration was assessed using the scratch wound assay described by Martinotti and Ranzato [94], with minor modifications. Both cell lines were seeded in 12-well plates at densities of 2.5 × 10^5^/mL and 3.0 × 10^5^ cells/mL, respectively, with fibroblasts forming a confluent monolayer after 24 h of incubation and keratinocytes after 72 h. Then, a single linear scratch was created across the monolayer using a sterile 10 µL pipette tip. The culture medium, containing detached cells, was carefully removed and replaced with fresh medium containing 2% (*v*/*v*) heat-inactivated FBS, with or without the essential oil at concentrations ranging from 25 to 100 µg/mL. Images of the wounded area were captured immediately after scratching (0 h) and after 18 h of incubation using an inverted light microscope (VisiScope IT417PH, VWR International, Radnor, PA, USA). Wound closure was quantified using ImageJ2 (version 2.16.0/1.54 g; Fiji distribution, Dresden, Germany), according to Suárez-Arnedo et al. [95]. The percentage of wound closure was calculated using the following equation:

$$Closed\;wound\;area\left(\%\right)=100-\left[\left(\frac{{OWA}_{18\;h}}{{OWA}_{0\;h}}\right)\times 100\right]$$

where ${\mathrm{OWA}}_{18\;h}$ represents the open wound area after 18 h of incubation and ${\mathrm{OWA}}_{0\;h}$ corresponds to the initial wound area at 0 h.

### 4.9. Statistical Analysis

Data analysis was performed using GraphPad Prism version 11 (GraphPad Software, San Diego, CA, USA), and results were expressed as mean ± standard error of the mean (SEM). Statistical comparisons among experimental groups were conducted using one-way ANOVA followed by Dunnett’s multiple comparisons test, considering the untreated control group as the reference. The homogeneity of variances was evaluated using the Brown–Forsythe test prior to ANOVA analysis. Differences were considered statistically significant when *p* < 0.05.

## 5. Conclusions

*L. gracilis* essential oil displays antifungal and antibiofilm activity against dermatophytes, including effects on established biofilms and potential relevance in the prevention of onychomycosis. These findings support its standardization in pharmaceutical formulations consistent with its traditional use in northeast Brazil. The essential oil disrupts fungal growth and biofilm formation at relatively low concentrations, while at sub-cytotoxic levels it is able to enhance cell migration, suggesting a potential role in promoting cutaneous wound healing.

These combined properties position *L. gracilis* essential oil as a candidate for inclusion in the Brazilian Pharmacopoeia Formulary of Phytotherapeutics, RENISUS (National List of Medicinal Plants of Interest to the Unified Health System—SUS), and official lists from the ‘*Farmácias Vivas*’ (Living Pharmacies) programs, which are community-based units that cultivate, process, and dispense medicinal-plant-based preparations.

Clinical efficacy, long-term safety, and the development of appropriate delivery systems supported by stability studies should be prioritized in future investigations to facilitate the integration of *L. gracilis* essential oil into herbal medicine–based strategies. In addition, future studies incorporating targeted mechanistic assays such as membrane integrity, ergosterol content, and ROS production assays, will be essential to fully elucidate the antifungal mechanisms underlying its activity.

## Figures and Tables

**Table 1:** Chemical composition of *L. gracilis* leaves essential oil from Fortaleza—Ceará, Brazil.

Compound	Relative Area (%)	RI^C^	RI^L^	MF
α-Pinene	0.5	939	939	C_10_H_16_
*p*-Menth-3-ene	0.29	978	974	C_10_H_18_
β-Pinene	0.39	1003	1002	C_10_H_16_
β-Terpinene	0.28	1018	1017	C_10_H_16_
*p*-Cymene	**12.13**	1029	1024	C_10_H_14_
*o*-Cymene	6.26	1035	1034	C_10_H_14_
γ-Terpinene	**9.29**	1063	1064	C_10_H_16_
Borneol	0.19	1171	1169	C_10_H_18_O
Thymol methyl ether	3.99	1239	1235	C_11_H_16_O
Thymol	**37.52**	1302	1302	C_10_H_14_O
δ-Guaiene	1.62	1440	1439	C_15_H_24_
α-Patchoulene	0.21	1451	1456	C_15_H_24_
Gurjunene	**10.95**	1494	1490	C_15_H_24_
Cubenol	0.27	1591	1592	C_15_H_26_O
Monoterpene hydrocarbons			29.14	
Oxygenated monoterpenes			41.70	
Sesquiterpene hydrocarbons			12.78	
Oxygenated sesquiterpenes			0.27	
Total Identified			84.00	

RI^C^: Calculated Retention Index; RI^L^: Retention Index from Literature; MF: Molecular Formula; HP-5MS column.

**Table 2:** Antifungal activity of *L. gracilis* (LG) essential oil against dermatophytes and *Candida* spp.

Strains	LG	
	MIC	MFC
*Epidermophyton floccosum* FF9	50	50
*Microsporum canis* FF1	50	100
*Microsporum gypseum* CECT 2908	50	200–400
*Trichophyton mentagrophytes* FF7	50	50–100
*Trichophyton mentagrophytes* var. *interdigitale* CECT 2958	100	400
*Trichophyton rubrum* CECT 2794	100	200–400
*Trichophyton verrucosum* CECT 2992	50	200–400
*Candida krusei* H9	800	>800
*Candida albicans* ATCC 10231	>800	>800
*Candida guilliermondii* MAT23	>800	>800
*Candida parapsilosis* ATCC 90018	>800	>800
*Candida tropicalis* ATCC 13803	400	800

Data are expressed in µg/mL (*n* ≥ 3 independent experiments). MIC: Minimum Inhibitory Concentration; MFC: Minimum Fungicidal Concentration. Value ranges represent the variation observed among repeated assays.

## Data Availability

The original contributions presented in this study are included in the article. Further inquiries can be directed to the corresponding author.

## Abbreviations

The following abbreviations are used in this manuscript:

DMSO	Dimethyl sulfoxide
ECM	Extracellular Matrix
EO	Essential Oil
GC–MS	Gas Chromatography–Mass Spectrometry
LG	*L. gracilis*
MIC	Minimum Inhibitory Concentration
MFC	Minimum Fungicidal Concentration
RENISUS	National List of Medicinal Plants of Interest to the Unified Health System (SUS)
SDA	Sabouraud Dextrose Agar
SUS	Unified Health System (Brasil)
YNB	Yeast Nitrogen Base
